# Acute effects of raisin consumption on glucose and insulin reponses in healthy individuals

**DOI:** 10.1017/jns.2013.33

**Published:** 2014-01-07

**Authors:** Amin Esfahani, Joanne Lam, Cyril W. C. Kendall

**Affiliations:** 1 School of Medicine, New York Medical College, Valhalla, NY, USA; 2 Clinical Nutrition and Risk Factor Modification Center, St Michael's Hospital, Toronto, ON, Canada; 3 Department of Nutritional Sciences, Faculty of Medicine, University of Toronto, Toronto, ON, Canada; 4 College of Pharmacy and Nutrition, University of Saskatchewan, Saskatoon, SK, Canada

**Keywords:** Raisins, Dried fruit, Glycaemic index, Glycaemic load, GI, glycaemic index, GL, glycaemic load, iAUC, incremental AUC, R20, raisins (20 g available carbohydrate), R50, raisins (50 g available carbohydrate), WB, white bread

## Abstract

Raisins are popular snacks with a favourable nutrient profile, being high in dietary fibre, polyphenols and a number of vitamins and minerals, in addition to being rich in fructose. In light of evidence demonstrating improvements in glycaemic control with moderate fructose intake and low-glycaemic index (GI) fruits, our aim was to determine the GI, insulin index (II) and postprandial responses to raisins in an acute feeding setting. A total of ten healthy participants (four male and six female) consumed breakfast study meals on four occasions over a 2- to 8-week period: meal 1: white bread (WB) (108 g WB; 50 g available carbohydrate) served as the control and was consumed on two separate occasions; meal 2: raisins (R50) (69 g raisins; 50 g available carbohydrate); and meal 3: raisins (R20) (one serving, 28 g raisins; 20 g available carbohydrate). Postprandial glucose and insulin were measured over a 2 h period for the determination of GI, glycaemic load (GL) and II. The raisin meals, R50 and R20, resulted in significantly reduced postprandial glucose and insulin responses when compared with WB (*P* < 0·05). Furthermore, raisins were determined to be low-GI, -GL and -II foods. The favourable effect of raisins on postprandial glycaemic response, their insulin-sparing effect and low GI combined with their other metabolic benefits may indicate that raisins are a healthy choice not only for the general population but also for individuals with diabetes or insulin resistance.

Raisins are one of the most commonly consumed dried fruits, are eaten across the globe, and have a unique nutrient profile that may confer distinctive health benefits when compared with other fruit. Raisins are a rich source of polyphenols and phenolic acids, which may serve as antioxidants and promote an anti-inflammatory environment with potential health benefits^(^^1^^–^^3^^)^. Raisins are also high in dietary fibre and prebiotics, such as inulin, which have been shown to produce a healthier colonic microflora profile in addition to possibly aiding weight management and reducing the risk of CVD^(^^4^^)^. A clinical study found that raisins as part of a healthy diet improved blood lipids and reduced other risk factors for CVD^(^^5^^)^.

Raisins are also high in fructose, which has a low glycaemic index (GI). While concerns have been raised that fructose may have adverse metabolic effects and promote weight gain, a recent meta-analysis^(^^6^^)^ demonstrated that moderate intakes of fructose may improve glycaemic control, without harming cardiometabolic risk factors^(^^6^^)^. This is especially important in light of recent evidence demonstrating that low-GI fruits may improve glycaemic and cardiovascular markers, including HbA1c and blood pressure^(^^7^^)^.

Given that raisins are the most commonly consumed dried fruit, are high in fructose and the controversy surrounding the cardiometabolic effects of fructose, we investigated the effect of raisins on postprandial glycaemia and insulinaemia in an acute feeding study.

## Methods

### Participants

Inclusion criteria included men or non-pregnant women aged 18–75 years who were in good health. Individuals with a known history of AIDS, hepatitis, diabetes or a heart condition, or individuals taking medication or with any condition that might make participation dangerous to the individual or affect the results were excluded.

A total of ten participants were studied. Using the *t* distribution and assuming an average CV of within-individual variation of incremental AUC (iAUC) values of 25 %, *n* 10 participants has 80 % power to detect a 33 % difference in iAUC with two-tailed *P* < 0·05.

### Protocol

The study was open-label with a partial randomised, cross-over design using standard GI methodology (ISO 26642:2010; International Organization for Standardization). Eligible participants were studied on four separate days over a period of 2–8 weeks with an interval of no less than 40 h and no more than 2 weeks between tests. On each test day, participants came to the clinic in the morning after a 10–14 h overnight fast. Participants were asked to maintain stable dietary and activity habits throughout their participation in the study. If any participant was not feeling well or had not complied with the preceding experimental conditions, the test was not carried out and was rescheduled for another day. On each test occasion participants were weighed, and two fasting blood samples were obtained by finger-stick at 5-min intervals. Finger-stick blood samples were collected from hands warmed with an electric heating pad for 3–5 min before each sample. Blood samples were collected into two separate vials: one (two or three drops of blood) for glucose analysis and the other (between six and eight drops of blood) for insulin. After the second fasting sample was collected the participant was provided with the test meal. At the first bite, a timer was started and additional blood samples were taken at 15, 30, 45, 60, 90 and 120 min. Before and during the test, a blood glucose test record was filled out with the participant's initials, identification number, date, body weight, test meal, beverage, time of starting to eat, time it took to eat, time and composition of last meal, and any unusual activities. During the 2 h test, participants remained seated quietly. After the last blood sample had been obtained participants were offered a snack and then allowed to leave.

The present study was conducted according to the guidelines laid down in the Declaration of Helsinki and all procedures involving human subjects/patients were approved by the Western Institutional Review Board^®^. Written informed consent was obtained from all participants before the start of the study.

### Study meals

Each participant participated in a total of four breakfast study meals. Two test meals were consumed: meal 1: R50, consisting of 50 g available carbohydrate from raisins; and meal 2: R20, consisting of 20 g available carbohydrate from raisins, which is one standard serving (28 g) of raisins. The control white bread (WB) meal, which provided 50 g available carbohydrate, was consumed twice. The macronutrient profiles of the study meals are provided in Table 1. The order of the test meals was randomised.

**Table 1.** Nutrient content of test meals

Test meal	Abbreviation	Amount (g)	Protein (g)	Fat (g)	Total CHO (g)	Dietary fibre (g)	Available CHO (g)
White bread	WB	108·0	9·3	0·8	52·0	2·0	50·0
Raisins (50 g CHO)	R50	69·0	1·7	0	53·4	3·4	50·0
Raisins (one serving)	R20	28·0	0·7	0	21·7	1·4	20·3

CHO, carbohydrate.

### Palatability

After consuming a meal, participants rated its palatability using a visual analogue scale anchored at very ‘unpalatable’ at one end (0) and ‘very palatable’ at the other (100). Therefore, the higher the number, the higher was the perceived palatability of the product.

### Blood samples

The finger-stick samples for glucose analysis were placed in a refrigerator and at the end of the test transferred to a –20°C freezer until analysed, which was performed within 5 d. A YSI model 2300 STAT analyser (YSI Life Sciences) was used for glucose analysis. For insulin analysis, the microvette tubes were centrifuged and the serum transferred to labelled polypropylene tubes and stored at –20°C before analysis. Insulin levels were measured using a Human Insulin ELISA Kit (Alpco Diagnostics).

### Data analysis

Data were entered into a spreadsheet by two different individuals and the values compared with assure accurate transcription. Incremental areas under the glucose and insulin response curves (AUC), ignoring area below fasting, were calculated. For the purposes of the AUC calculation, fasting glucose and fasting insulin were taken to be the mean of the first measurement of the blood glucose concentrations and serum insulin concentrations at times –5 min and 0 min. The GI and insulin index were calculated by expressing each participant's AUC for the test food as a percentage of the same participant's mean AUC for the two white bread controls. Values >2 sd above the mean were excluded. The blood glucose and serum insulin concentrations at each time, AUC, GI and insulin index values were subjected to repeated-measures ANOVA examining for the main effects of test meal and the meal × participant interaction. After demonstration of significant heterogeneity, the significance of the differences between individual means was assessed using Tukey's test to adjust for multiple comparisons. Means differing by more than the LSD (least significant difference) were statistically significant, two-tailed *P* < 0·05.

Glycaemic load (GL) was calculated using the formula: GL = GI × g of available carbohydrate in the portion.

### Glycaemic index classification

Using the classification of Brand-Miller for the glucose scale, products with a GI of 55 or lower are classified as being low GI; those with a GI of 56 to 69 are classified as medium, while those with a GI of 70 or greater are classified as high GI.

## Results

A total of ten participants (four male and six female) with a mean age of 39 (sd 11) years and an average BMI of 26·4 (sd 6·2) kg/m^2^ completed the study.

### Within-subject variation of reference food

The mean within-subject CV of the iAUC values after the two repeated WB tests was 17·0 ± 3·6 % and was thus considered technically satisfactory (average intra-subject variation of less than 30 %).

### Palatability

Palatability scores are presented in Table 2. The subjective palatability of the R50 and R20 meals was higher than that of the WB control. However, this difference did not reach statistical significance.

**Table 2.** Palatability, glycaemic index (GI), GI category, glycaemic load (GL), GL category and insulin index (Mean values with their standard errors)

		Palatability (mm)		GI					Insulin index	
Test meal	Abbreviation	Mean	sem	Mean	sem	GI category*	GL	GL category	Mean	sem
White bread	WB	63·0^a^	10·0	71·0^a^		High	35·5^a^	High	71·0^a^	
Raisins (50 g CHO)	R50	75·0^a^	6·0	49·0^b^	4·0	Low	24·5^b^	N/A	38·0^b^	3·0
Raisins (one serving)	R20	72·0^a^	6·0	N/A		N/A	9·9^c^	Low	N/A	

CHO, carbohydrate; N/A, not applicable.

^a,b,c^ Mean values within a column with unlike superscript letters were significantly different (*P* < 0·05).

* Category from GI Factor (Atkinson *et al.*^(^^27^^)^).

### Postprandial glucose response and glycaemic index

**Fig. 1.**
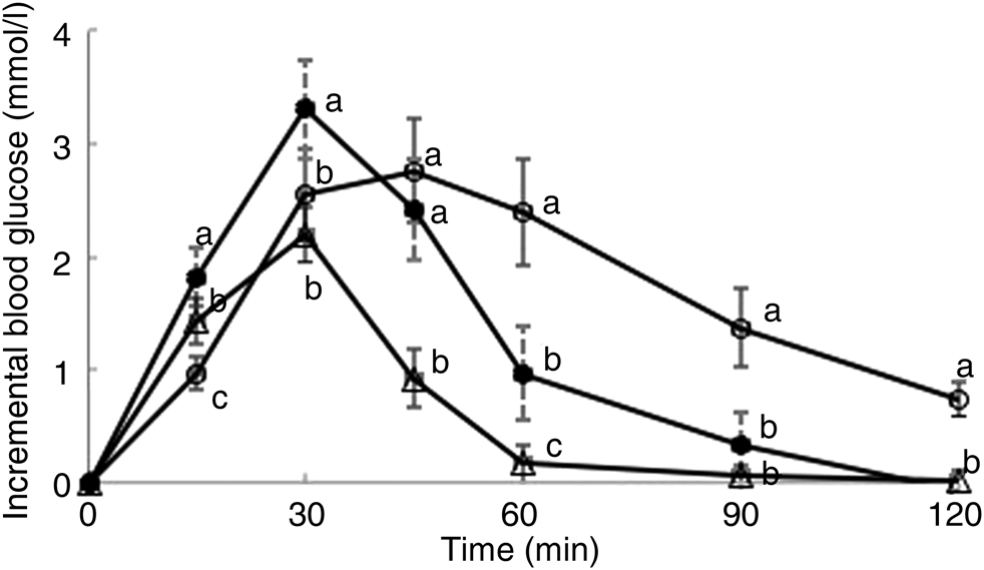
Postprandial glucose responses to three meals containing 50, 50 and 20 g of available carbohydrates from white bread (○), raisins (•) and raisins (∆), respectively. Values are means, with standard errors represented by vertical bars. ^a,b,c^Mean values at a specific time point with unlike letters were significantly different (*P* < 0·05).

**Fig. 2.**
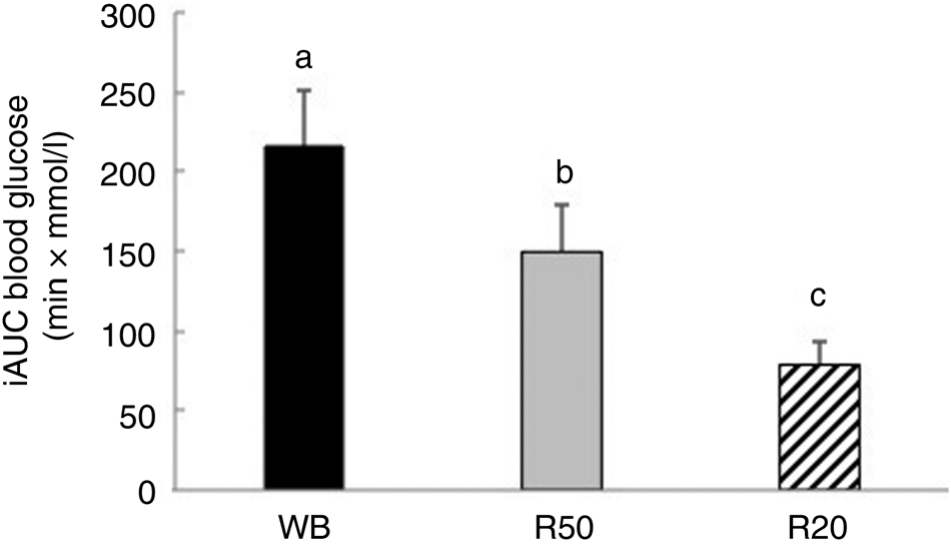
Incremental AUC (iAUC) for glucose after consumption of three meals containing 50, 50 and 20 g of available carbohydrates from white bread (WB), raisins (R50) and raisins (R20), respectively. Values are means, with standard errors represented by vertical bars. ^a,b,c^Mean values with unlike letters were significantly different (*P* < 0·05).

Postprandial incremental glucose levels after the R50 meal were significantly higher than those after the WB meal at 15 and 30 min. At 60, 90 and 120 min, however, the postprandial incremental glucose levels after R50 were significantly lower than after WB (Fig. 1). iAUC were significantly lower after both raisin meals than after WB (Fig. 2). The final GI and GL values are presented in Table 2.

### Postprandial insulin response and insulin index

**Fig. 3.**
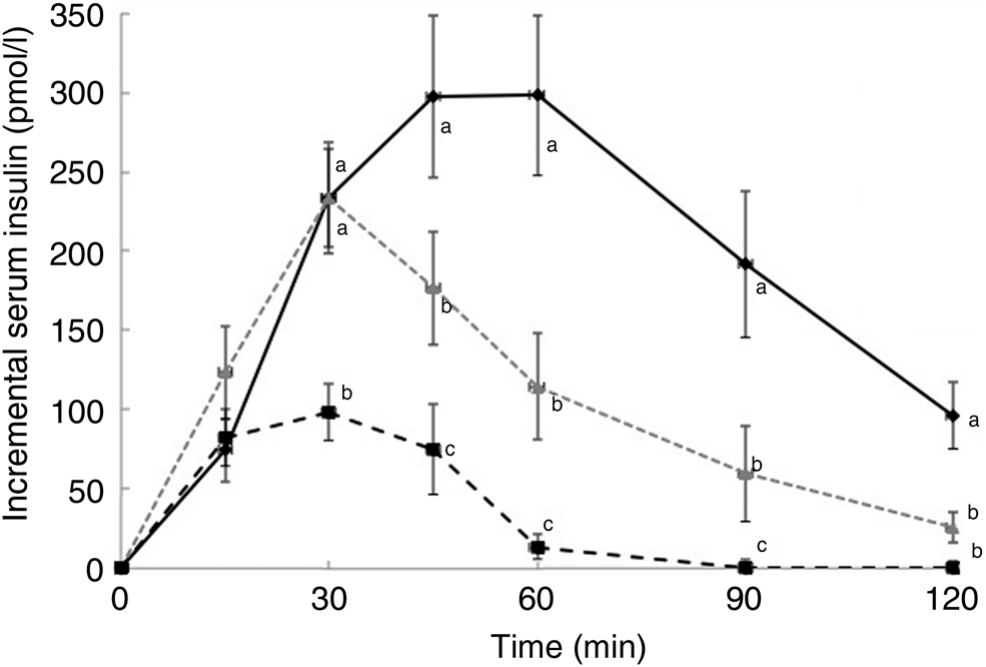
Postprandial insulin responses to three meals containing 50, 50 and 20 g of available carbohydrates from white bread (♦), raisins (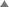) and raisins (■), respectively. Values are means, with standard errors represented by vertical bars. ^a,b,c^Mean values at a specific time point with unlike letters were significantly different (*P* < 0·05).

**Fig. 4.**
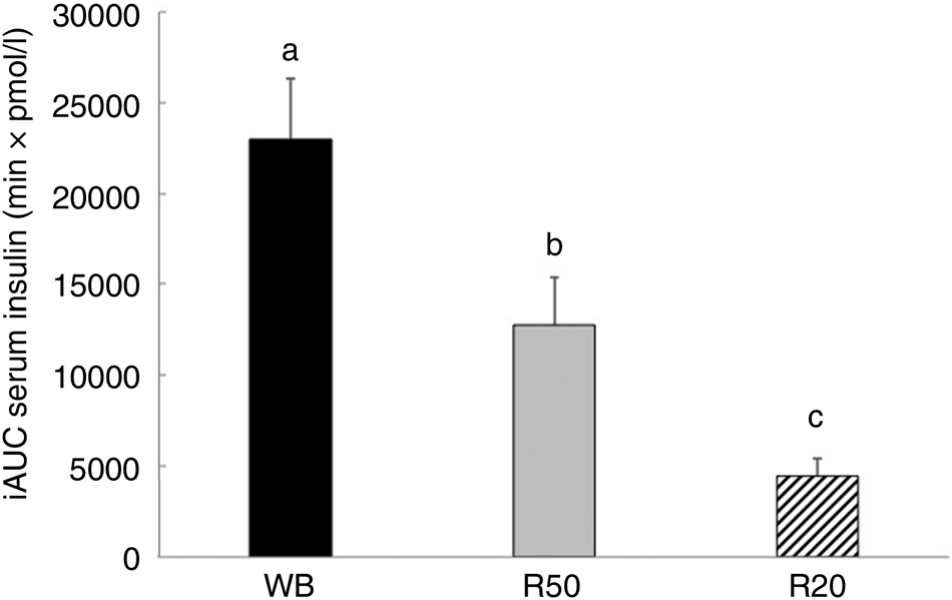
Incremental AUC (iAUC) for insulin after consumption of three meals containing 50, 50 and 20 g of available carbohydrates from white bread (WB), raisins (R50) and raisins (R20), respectively. Values are means, with standard errors represented by vertical bars. ^a,b,c^Mean values with unlike letters were significantly different (*P* < 0·05).

There was no significant difference between the WB and R50 meals in incremental postprandial insulin levels at 15 and 30 min. However, insulin levels were significantly lower at 45, 60 90 and 120 min with the R50 meal compared with WB (Fig. 3). iAUC were also significantly lower with raisins compared with the WB control (Fig. 4). The final insulin index values are presented in Table 2.

## Discussion

The present study demonstrates that raisins are a low-GI and -insulin index fruit that provides a favourable postprandial glucose and insulin response. In terms of postprandial glucose response, raisins elicited a swifter response compared with WB for the first 30 min. This, however, was followed by a sharp decline and an overall lower AUC for glucose when compared with WB (*P* < 0·05), which is commonly observed with other fruits. This postprandial glucose response pattern may be explained by the high sucrose content of raisins. The sucrose would be rapidly digested and the glucose rapidly absorbed relative to starch. However, the fructose, which is responsible for 50 % of the available carbohydrate content of raisins, would not contribute to the rise in blood glucose. Evidence from other studies suggests that the benefits of fructose on glycaemic control may extend beyond simple replacement of glucose. Moore *et al.*^(^^8^^)^ demonstrated that the addition of only 7·5 g of fructose, levels which are slightly lower than the fructose content of one serving of raisins, to 75 g of glucose as part of an oral glucose tolerance test significantly lowered the glucose response when compared with 75 g glucose with no added fructose^(^^8^^)^. A potential mechanism of action for this improved glycaemic response with fructose ingestion may be enhanced hepatic glucose uptake. Fructose ingestion increases the hepatic concentrations of fructose-1-phosphate (first product of hepatic fructose metabolism), which in turn competes with fructose-6-phosphate for binding to glucokinase regulatory protein (GKRP). This leads to the release of glucokinase (rate-limiting enzyme in the hepatic metabolism of glucose) from GKRP, causing hepatic metabolism and further uptake of glucose and thus lower postprandial glucose concentrations^(^^8^^,^^9^^)^. This glycaemic advantage with moderate intakes of fructose over glucose is not a novel finding, and has been reported in both healthy individuals and patients with diabetes in the 1970s and 1980s^(^^10^^–^^12^^)^. A recent meta-analysis put this link into perspective by demonstrating that small doses of fructose < 10 g/meal or < 36 g/d can significantly improve serum levels of HbA1c and fasting glucose levels^(^^6^^)^. Furthermore, this daily intake level was not associated with any adverse metabolic effects that have been linked to high intake of fructose such as dyslipidaemia^(^^6^^)^.

Also of interest is the low GI of raisins as determined by the present study (49 based on the glucose scale). A previous study by Jenkins *et al.*^(^^13^^)^ reported the GI of raisins to be 64. However, this study was conducted on only six subjects. More recently a study by Kim *et al.*^(^^14^^)^ reported GI values of 49 in sedentary individuals, 49 in individuals with prediabetes and 55 in aerobically trained adults. These results are very similar to the GI value determined for raisins in the present study. The health benefits of low-GI fruit were demonstrated in a recent secondary analysis of a clinical intervention that showed that low-GI fruit consumption as part of a low-GI diet was associated with statistically significant reductions in HbA1c, systolic blood pressure and overall CHD risk^(^^7^^)^. The original randomised clinical trial assessed the effects of a low-GI *v.* a high-fibre diet on glycaemic control in patients with type 2 diabetes and included fruit intake advice as part of the dietary intervention^(^^15^^)^. The secondary analysis included 152 patients and demonstrated that the GI of fruit was an independent predictor of HbA1c reduction and that the lowest quartile of GI intake led to the greatest reduction in HbA1c^(^^7^^)^. It is important to note that in this study grapes were considered high-GI foods (GI > 90 based on the bread scale). However, the present study suggests that raisins have a low GI (GI < 70 based on the bread scale). The present study also demonstrated that both serving sizes of raisins studied (69 and 28 g) are low-GL foods. The beneficial effects of low-GI and -GL foods on diabetes and risk of CVD have been demonstrated by a number of large cohort studies^(^^16^^–^^18^^)^. Lastly, the type and amount of fibre present in raisins should not be overlooked as another component that may account for the lowered glycaemic response. Overall, the present findings support the notion that incorporation of raisins as part of a healthy, low-GI diet in patients with diabetes or impaired glucose tolerance can potentially improve glycaemic management and provide additional cardiovascular benefits.

The present study also demonstrated that raisins lead to a lower postprandial insulin response when compared with WB. This insulin-sparing effect may also be in part due to the fructose content of raisins. Fructose is not an insulin secretagogue and, unlike glucose, does not require insulin for cell entry^(^^19^^)^. The insulin-sparing effects of fructose have been demonstrated in a number of other studies^(^^20^^–^^22^^)^. While long-term impacts of raisins on insulin control require further investigation, the present study suggests that raisins, through acute postprandial insulin-sparing effects, may be a healthy food choice in patients with insulin resistance or diabetes.

The major limitation of the present study, as with all acute feeding studies, is the inability to translate these acute findings to long-term benefits. However, at least in terms of the beneficial effect of fructose on glycaemic management, previous studies have shown that these effects are sustainable over a longer period of time^(^^23^^,^^24^^)^. Another shortcoming is the sample size. While the use of ten subjects has been validated by a number of studies, nevertheless this sample size reduces the study precision and may lead to exaggerated associations.

While the potential benefits of moderate consumption of fructose on glucose control have been overshadowed by the adverse outcomes, especially on serum lipids^(^^23^^,^^25^^,^^26^^)^, associated with overconsumption and over-utilisation of high-fructose corn syrup in the everyday diet, the benefits of fructose as a component of whole fruits should not be overlooked. Raisins are popular snacks that are readily accessible at a reasonable price. Their nutrient profile, being high in antioxidants, dietary fibre, prebiotics, vitamins and minerals, indicate that they could contribute to overall health. While long-term studies are needed, the present study demonstrates that in addition to the aforementioned benefits, raisins can acutely improve postprandial glycaemic control and, as a low-GI food, may serve as a healthy snack, when used in moderation, in the diets of healthy individuals and for those with diabetes or impaired glucose tolerance.
